# The Adaptive Power of *Ammophila arenaria*: Biomimetic Study, Systematic Observation, Parametric Design and Experimental Tests with Bimetal

**DOI:** 10.3390/polym13152554

**Published:** 2021-07-31

**Authors:** Tarciana Araújo Brito de Andrade, José Nuno Dinis Cabral Beirão, Amilton José Vieira de Arruda, Cristina Cruz

**Affiliations:** 1 Centro de Investigação em Arquitetura, Urbanismo e Design (CIAUD), Faculdade de Arquitetura, Universidade de Lisboa, 1349-063 Lisboa, Portugal; jnb@fa.ulisboa.pt; 2 Programa de Pós-Graduação em Design (PPGDesign), Universidade Federal de Pernambuco, Recife 290, Brazil; amilton.arruda@ufpe.br; 3 Department of Plant Biology, Faculdade de Ciências da Universidade de Lisboa, 1749-016 Lisboa, Portugal; ccruz@fc.ul.pt

**Keywords:** biomimetic and design, climate adaptation, *Ammophila arenaria*, reversible leaf movement, bimetal

## Abstract

The aim of our study was to apply a biomimetic approach, inspired by the *Ammophila arenaria*. This organism possesses a reversible leaf opening and closing mechanism that responds to water and salt stress (hydronastic movement). We adopted a problem-based biomimetic methodology in three stages: (i) two observation studies; (ii) how to abstract and develop a parametric model to simulate the leaf movement; and (iii) experiments with bimetal, a smart material that curls up when heated. We added creases to the bimetal active layer in analogy to the position of bulliform cells. These cells determine the leaf-closing pattern. The experiments demonstrated that creases influence and can change the direction of the bimetal natural movement. Thus, it is possible to replicate the *Ammophila arenaria* leaf-rolling mechanism in response to temperature variation and solar radiation in the bimetal. In future works, we will be able to propose responsive facade solutions based on these results.

## 1. Introduction

There are different nomenclatures to designate human activities concerning nature as biomimetic, bionic, and biomimicry [1,2,3]. For this study, we will focus on biomimetic. Otto H. Schmitt created the term biomimetic in the 1960s. In doing so he intended to distinguish knowledge from biophysics and highlight nature’s potential for innovation [3]. Biomimetic is the transdisciplinary language that connects project designers to biologists [4] and technology [3]. It allows us to develop analogies with nature to solve architectural and technical problems [5,6] and design issues.

Current knowledge demonstrates the expansion of adaptable and responsive facade propositions related to biomimetic approaches [2] and materials research [5]. These structures use analogies to biological systems and their response capacity is rooted in the material itself [5,7]. By reducing mechanical system usage and power consumption it is possible to increase their responsiveness to environmental conditions [2]. The shape-changing smart materials are used to develop kinetic facades that adapt to different climatic conditions [8]. The most frequently used materials are SMA—Shape Memory Alloys—and wood-based bio-composites; followed by bimetals, electroactive polymers, composite bimetals, shape memory polymers, and hydrogels [8].

In turn, parametric design is based on the exploration and re-editing of associative relationships to generate the logical construction of geometry [9]. Parametric systems can explore performance-based design [5,10] to develop optimized facade solutions for shading [5,11], daylighting, thermal comfort, visual comfort, energy generation, life-cycle analysis, and energy efficiency [12].

This work aims to analyze the adaptive response of *Ammophila arenaria* (*A. arenaria*) leaves through the problem-based biomimetic methodology [4,13,14,15]. To do so, we first identified our reference study object. Then, we carried out a set of two observational studies that will be described in depth in Section 2.1.3, 3.1 and 3.2.2. We developed experiments with bimetals that curl up in response to rising temperatures [16,17].

The present study is part of ongoing research, whose objective is to use a biomimetic approach to create adaptable and responsive cobogós based on a behavioral and morphological analogy with plant movements. A Cobogó is a breeze block—a symbol of modern Brazilian architecture—which had its production process patented in 1929 in Recife-PE [18]. The cobogó’s functionality is to ventilate, illuminate, filter the incidence of sunlight, and promote privacy for internal spaces [19]. Artifacts similar to cobogós are found in several parts of the world under different production processes and nomenclatures [20]. In the future, we intend to propose a cobogó algorithm to produce adaptive and responsive facades to provide shading without using electromechanical devices, promoting sustainability.

## 2. Materials and Methods

### 2.1. Biomimetic Study

There are two main methodological processes encompassing different terminologies for biomimetic approaches [4,13,14,15]: (a) Solution-based (bottom-up): the observation of nature leads to a technological design, and (b) Problem-based (top-down): seeks a solution in nature to a specific engineering problem.

**Figure 1:**
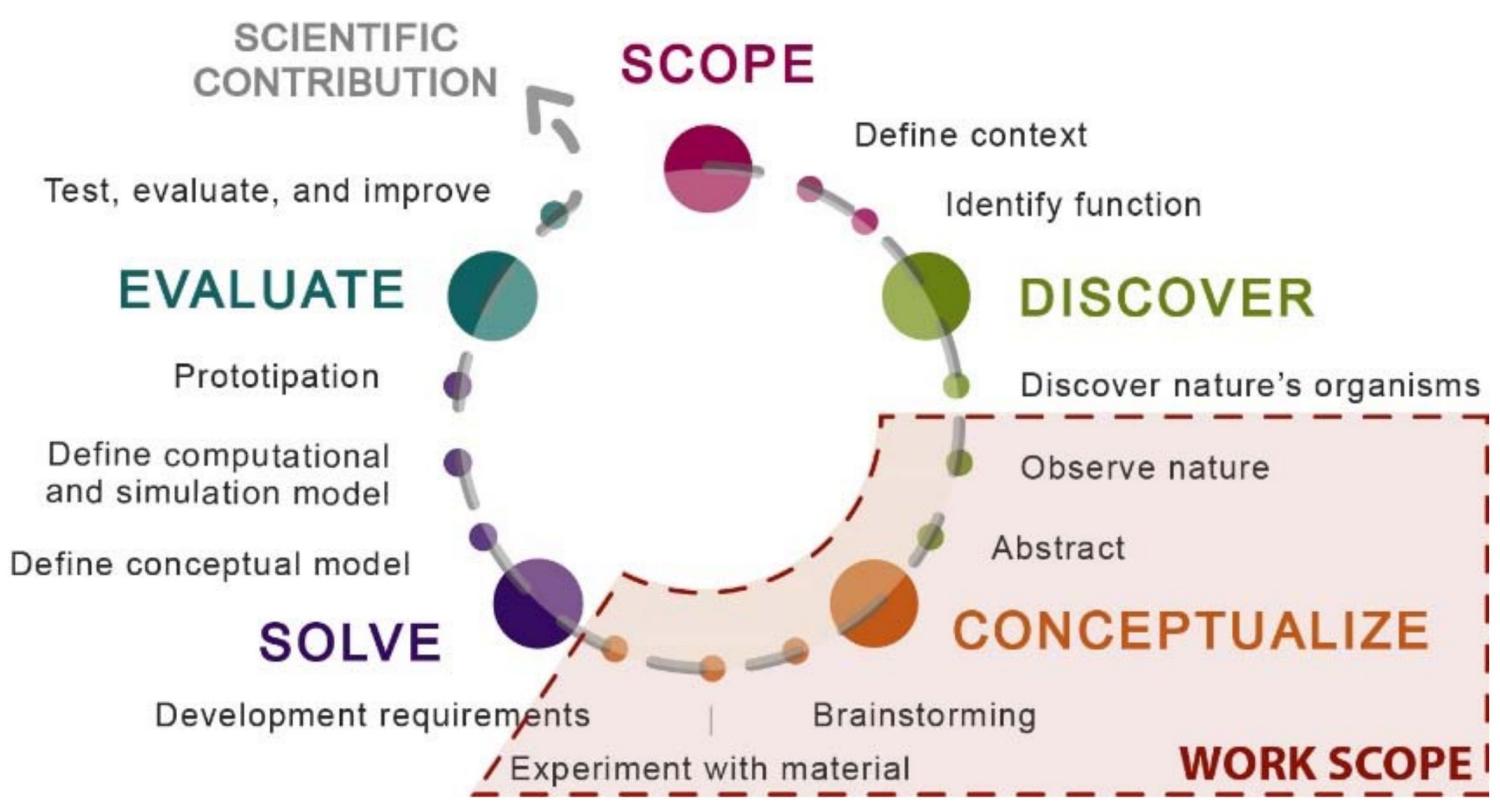
Research methodology. Source: Authors.

In this work, we used the problem-based biomimetic methodology to analyze the adaptive response of *Ammophila arenaria* leaves and guide the transfer of biological principles to architecture [4,14,15] (Figure 1).

#### 2.1.1. Scope and Discover

To rethink the Cobogó, we will adopt the climatic context of the Municipality of Lisbon in Portugal as a reference. The area has hot, dry summers and mild winters [21]. Lisbon has a temperate climate, strongly influenced by the Gulf Stream [4,22]. The maximum and minimum average annual temperatures are 21 °C and 13.7 °C, respectively [21].

We specify the group of plants that possess nastic movement [23] as reference organisms for the biomimetic approach to research. Nastic is the plants’ kinetic reaction in response to environmental stimuli regardless of direction [5,24]. The direction of the nastic movements is dictated by intrinsic asymmetries in the plants’ anatomy [25].

**Figure 2:**
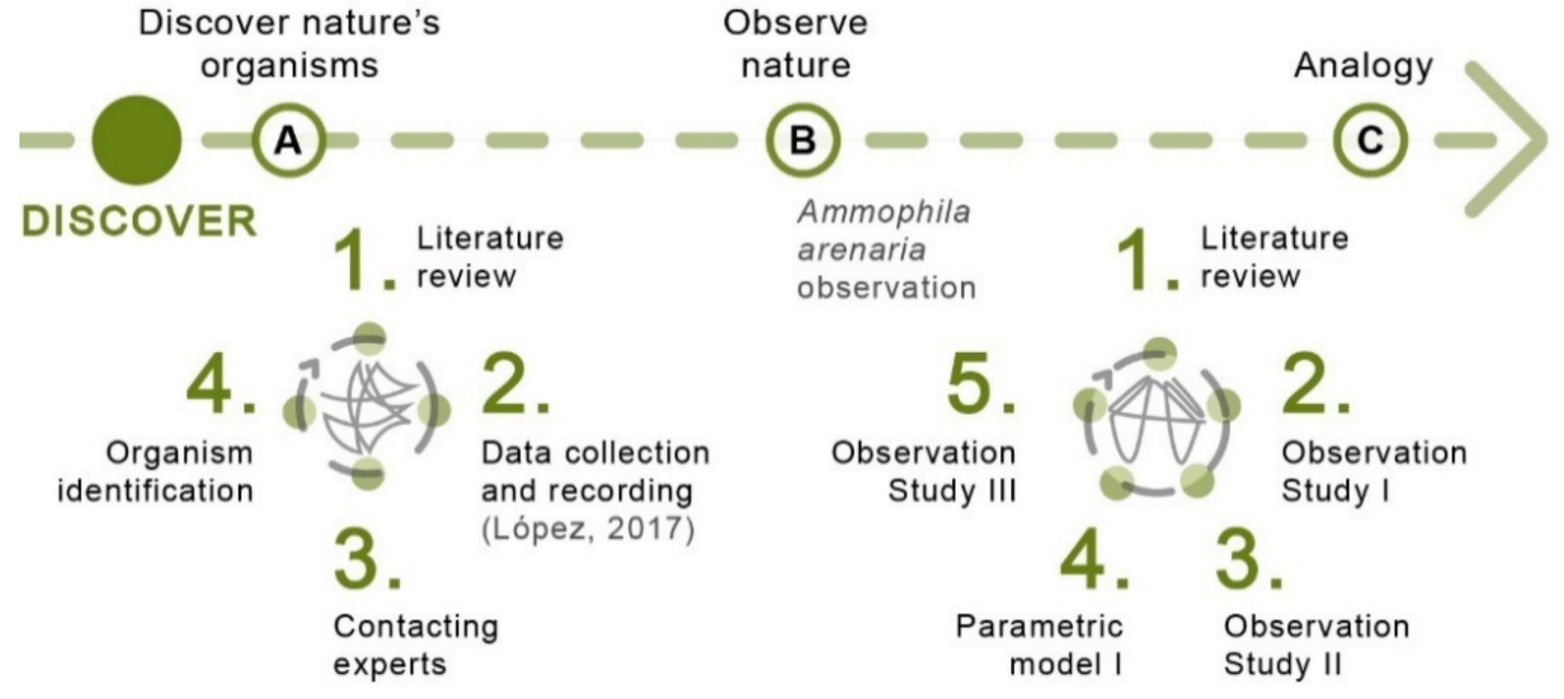
Phases in the research methodology. Source: Authors.

During the “Discovering nature’s organisms” stage, we established the following criteria to select the reference organism: (a) possess nastic movement; and (b) be accessible in Lisbon, to enable observational studies. Our literature review [23] identified and classified the different types of plant nastic movements and the organisms’ strategies (Figure 2).

Professional biologists supported us in finding an organism compatible with our criteria and available in the Portuguese flora. They suggested a few organisms, and after extensive research, we identified the *Ammophila arenaria* as a relevant biological system. We selected *A. arenaria* as the study object of the present work because it meets the two established selection criteria: reversible leaf movement [26,27] classified as hydronastic movement (Section 2.1.2) and easy accessibility in Lisbon [28,29].

This paper covers the literature review, observation studies, and analogy of *Ammophila arenaria* strategies in the discovery phase (Figure 2). Furthermore, we demonstrate our experimentation process with the bimetal.

#### 2.1.2. Observe Nature: Organisms Characteristics

*Ammophila arenaria* is the scientific name for marram grass, its profusion along the shoreline of Western Europe and Northern Africa is remarkable and demonstrates the importance of this organism for the fixation of coastal dunes [27,30]. The *A. arenaria* is an angiosperm belonging to the *Poaceae* family. It is a perennial, rhizomatous grass and reaches up to 120 cm in height, which forms dense tufts [27]. The leaves are pointed, rigid and unrolled, around 6 mm wide [31] and 60–90 cm long [27].

**Figure 3:**
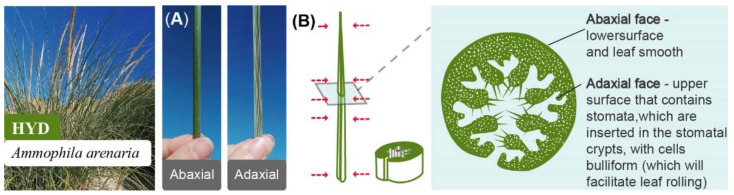
Characteristics of *A. arenaria*. (**A**,**B**) abaxial and adaxial face. Source: adapted by [13].

*Ammophila arenaria* leaves are usually well rolled, except when under humid environmental conditions [27]. The lower epidermis of the leaf or the abaxial face (Figure 3A) is smooth and greyish-green. The upper epidermis or adaxial face (Figure 3A) is ribbed and densely covered by tiny trichomes [27,31] (Figure 3).

**Figure 4:**
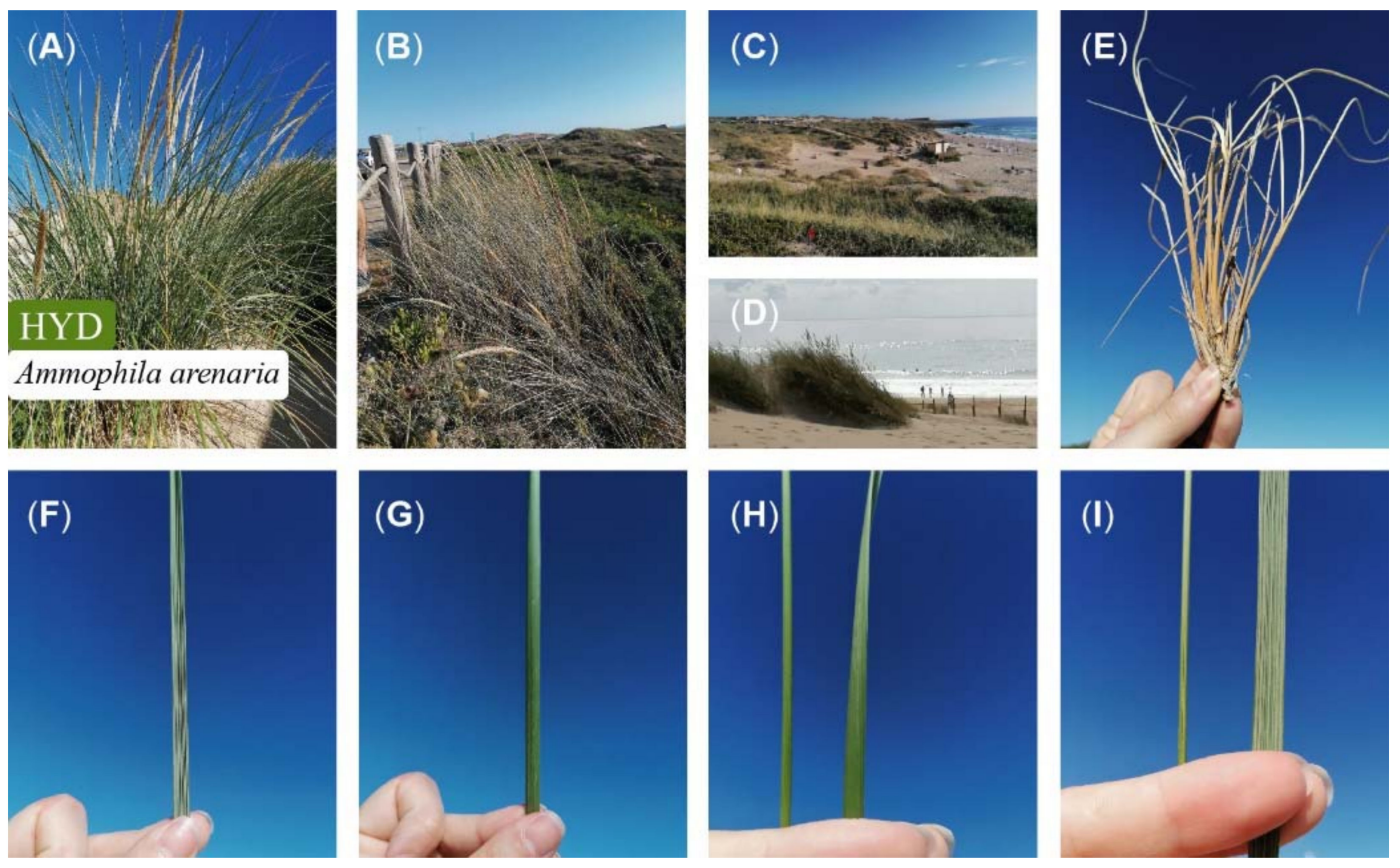
Photographic record of *A. arenaria*. (**A**–**D**) marram grass; (**E**) general form; (**F**) adaxial face and (**G**) abaxial leaf face; (**H**,**I**) comparison between a completely rolled leaf and an unrolled leaf. Source: Author.

In Portugal, *Ammophila arenaria* subsp. Arundinacea is found throughout the continental coastal strip, known as estorno [29]. The Portuguese populations of *A. arenaria* subsp. Arundinacea have erect and robust stems, reaching between 50–120 cm high. The leaves of this plant measure about 1.5–2 mm in diameter [28] (Figure 4).

The *Ammophila arenaria* leaf anatomy is quite sophisticated, it enables the adaptation to water and salt stress [26], which triggers the reversible leaf movement. Its functioning perfectly reflects such adaptations as the leaf-rolling mechanism. This mechanism corresponds to the typology of a nastic movement—the hydronastic movement—that reduces transpiration, leaf dehydration, and light interception [32]. This movement provides osmotic adjustment and protection against the effects of excessive radiation [26]. This movement is commonly found in organisms of the *Poaceae* family (grass) [33].

**Figure 5:**
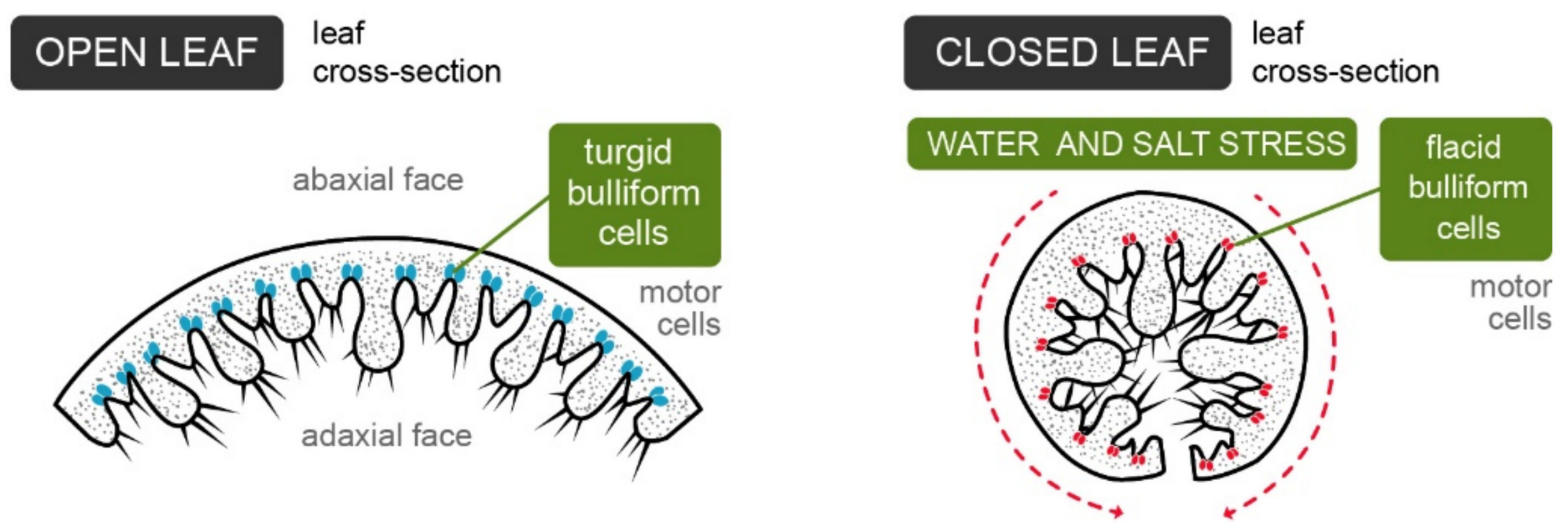
*Ammophila arenaria*’s leaf-rolling mechanism. Source: Authors.

The presence of bulliform cells is critical to facilitate the folding and rolling of leaves [26,34]. They are in the adaxial face of *A. arenaria,* at the base of stomatal crypts—which are grooves between the ribs of the upper epidermis surface that travel the entire length of the leaf [26]. These cells are large, have a thin outer wall, and have no cuticle [33]. The absence of cuticles allows these cells to respond to turgidity by speeding up transpiration. Bulliform cells act as a motor, they retract the cell’s volume due to water loss, causing the leaf to roll or fold [26,33]. In humid conditions, the cells are turgescent, making the leaves unroll [26] (Figure 5). The position of the bulliform cells in the epidermis determines the leaf closing pattern [33].

The movement induced by bulliform cells reduces the transpiration and decreases the leaf surface area [34] and contains the perspired water vapor [26]—preventing moisture loss to the environment. The stomata are pores necessary for the gas exchange of plants. When the leaf-rolling mechanism takes place, the stomata close reducing transpiration. Due to a decrease in surface area, the leaf rolling also reduces the abrasive impact of sand grains on leaf tissues [26].

The adaxial side is ribbed and densely covered by tiny hairs that protect the stomata. They contribute to reducing gas exchange in the plant and expanding the water vapor diffusion path [27].

The abaxial face is devoid of stomata [26], which contributes to reducing transpiration, combined with the cuticle’s thickening. This cuticle is thick and clear. It increases the plant’s reflection coefficient, protecting it from radiation [26]. The cuticle is also hydrophobic, as it provides a waxy coating preventing water loss.

#### 2.1.3. Observe Nature: Systematic Observation

There are three categories to identify the levels of plant adaptation [13,14]: (i) physiological (responses through chemical processes and physiological characteristics); (ii) behavioral (an organisms’ actions in order to survive. This category marks an interaction between the organism and its environment), and (iii) morphological (an organisms’ size, shape, and pattern, surface segmentation trends, or structure depending on the climatic environment).

We adopted the observation process to explore and understand the *Ammophila arenaria*’s morphology and behavior. We carried out two observation studies. The first analyzed the reversible movement of the leaf. In the second study, we analyzed the morphology of *A. arenaria* cross-sections—specifically the location of the bulliform cells in the stomatal crypts and its role in triggering the nastic movement.

##### Observation Study I

The first observation study aims at identifying the morphological and behavioral aspects of the reversible movement of the samples under analysis. For such a goal, we analyzed the leaf movement by dehydration and rehydration in three stages: (a) sample preparation, (b) dehydration cycle, and (c) rehydration cycle.

**Figure 6:**
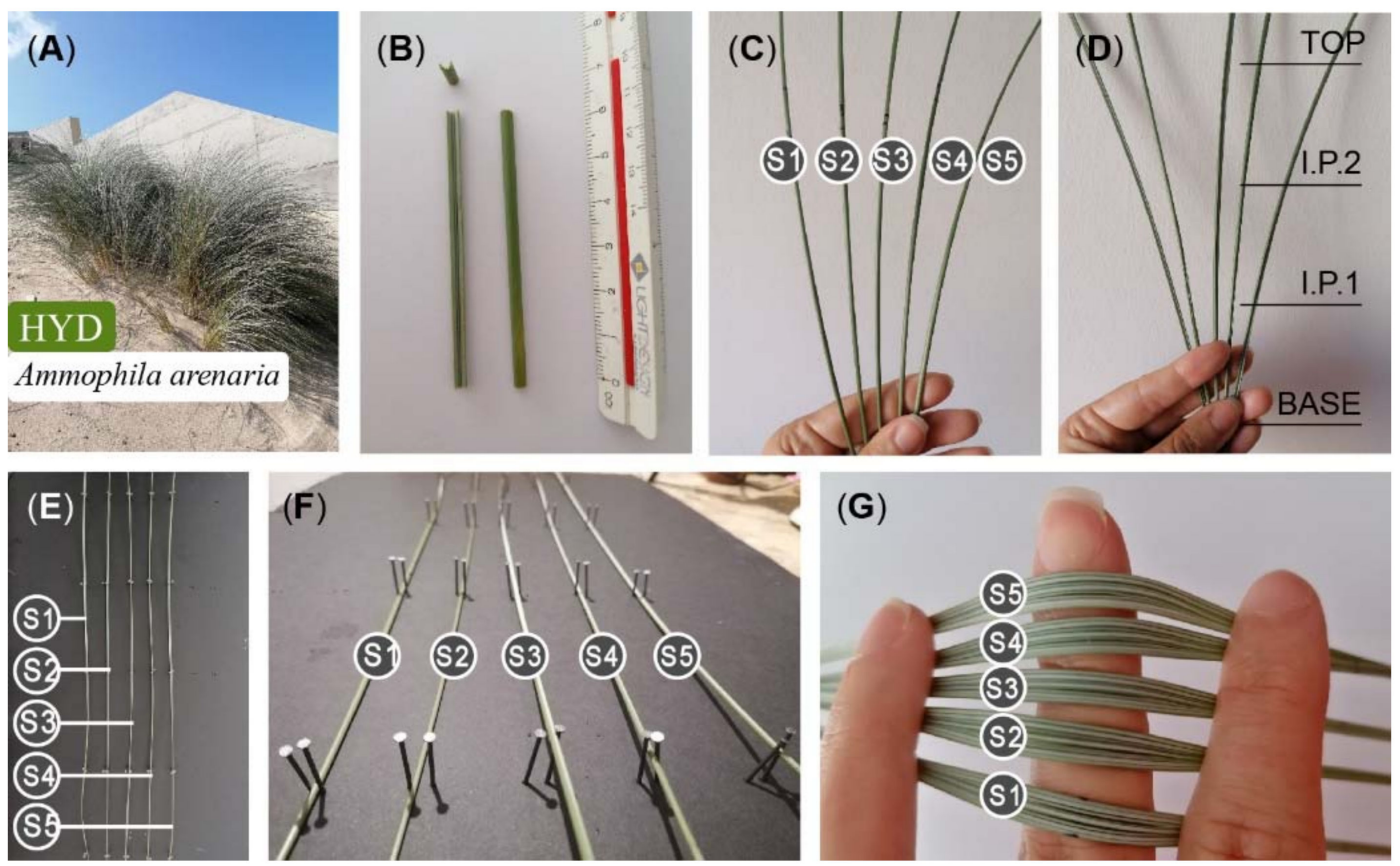
(**A**) Photographic register of *Ammophila arenaria*; (**B**) reference of leaf dimensions of marram grass; (**C**) adaxial face of the leaf samples; (**D**) abaxial face with identification of the four measurement points; (**E**,**F**) positioning of the samples in a platform that allows flexible setting; (**G**) adaxial face. Source: Authors.

For sample preparation, we collected leaf samples of *A. arenaria*, at Praia do Guincho, in Cascais, in Lisbon, Portugal (38°43′48.0″ N 9°28′25.0″ W) on 28 September 2020. We then selected five samples and hydrated them in drinking water for twenty hours. On 29 September, we registered their heights and four distinct points on each sample: base, intermediate point 1 [I.P. 1], intermediate point 2 [I.P. 2], and top (height < 10 cm) (Figure 6).

For each point, we recorded the Dehydration Cycle, which took place between 10:00 and 13:00. We positioned the samples on a platform that allowed us to observe their movement, without constraints. We registered the width of each sample at the four pre-defined points every thirty minutes in an Excel spreadsheet. The samples should vary in width and were measured using a caliper and photographed.

The Rehydration Cycle occurred between 13:00 and 16:30. We placed the bases of the samples in drinking water and measured the extent of their opening at 13:00. We re-inserted each sample in an aqueous medium immediately after measuring them. This process happened systematically, every thirty minutes, until 16:30. Twenty-four hours after the beginning of the experiment, we re-measured all the samples.

Thus, during this observational study, we identified the dimensions and width variation to each point observed. As a result, we identified average dimensions, the response time for movement, and the leaf region with the most significant width variation.

##### Observation Study II

The second study aims at identifying the morphological aspects of *Ammophila arenaria* to create a crease pattern from the location of the bulliform cells in the stomatal crypts. The position of these cells is relevant in determining the leaf closure pattern [33]. This study arises in three stages: (a) cross-sections selection; (b) cross-sections analysis with a qualitative synthesis production; and (c) creation of crease patterns inspired by the *A. arenaria*.

For cross-section selection, we analyzed the morphology of the cross-sections of *A. arenaria* from images available on the Science Photo Library website [35]—a scientific and medical image and video bank. To protect intellectual property and image rights, the synthesis of the analysis of the cross-sections shall credit the proper authors. It will present the image codes available on the website [35].

We established the following criteria for selecting cross-sections: (a) readability; (b) should be fully framed; (c) should be from different leaves, and (d) present similar levels of opening and closing. Four of the nine cross-sections found in the data collection met our criteria and were used to make up the corpus of our analysis. We will explain the stages of cross-section analysis and crease patterns synthesis in the results section.

#### 2.1.4. Conceptualize

Humidity control is an inherent characteristic of *Ammophila arenaria*, and research suggests approaches for responsive facade elements that adopt materials with hygroscopic properties are in demand. The performance of these materials is enhanced by hot and humid climatic contexts, cooling down built spaces. The effectiveness of hygroscopic materials is minimized in cold or dry environments due to little evaporation.

Given the bioclimatic perspective of buildings in Portugal, the climate is an essential variable for design conception and the interaction between the sun and buildings are a fundamental part of the design process [36]. Solar radiation and outside air temperature are the climatic variables that most influence heat transfer in buildings [36].

Bearing this in mind, at the beginning of the conceptualization stage, we carried out a focus group to rethink the cobogó for Lisbon’s climate using the *A. arenaria* as an inspiration [37]. The results revealed that sun protection and lighting are seen as a conceptual reference for thermal control of the facade using shade [37]. Such parameters gained strength in detriment to controlling natural ventilation and humidity. The idea of allowing the user to control ventilation was also mentioned. Therefore, we chose to explore the development of an adaptable and responsive cobogó to promote shading in the built spaces to contribute to the control of heat gain and loss.

In analogy to the reversible behavior of *Ammophila arenaria* leaves, the shape-changing materials [8] emerged as an option to use in our experiments. We selected the bimetal [10,16,17,38] as reference material for this end, it behaves similarly to *A. arenaria*. Its adaptive behavior induces the leaves to close when exposed to adverse environmental conditions and reopen as these conditions are eased.

The bimetal has different nomenclatures, such as thermobimetal and thermostatic bimetal. This material tends to curve when heated, acting as a “trigger” in a kinetic response to the increase in temperature on the surface [16]. The explanation for such behavior lies in its composition, which consists of at least two layers of metal with different thermal expansion coefficients [39].

**Figure 7:**
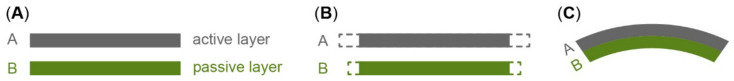
(**A**) Bimetal consists of two layers of metal with different rates of thermal expansion; (**B**) these metals, when heated, expand differently; (**C**) such layers are permanently linked to each other. As the passive layer limits the active layer, the bimetal compound follows a deflection curve when heated. Source: adapted from [16].

The layer with the highest expansion coefficient is called active [39,40] and usually consists of an alloy containing nickel, iron, manganese, or chromium in different quantities [38]. The layer with the lowest coefficient is called passive [39,40], or invar [17]. The passive layer is often composed of an iron-nickel alloy containing 36% nickel [38]. The temperature variation will cause the active component to expand more than the passive, causing deflection. In the absence of external forces, the bimetal will typically take the form of an arch [38] (Figure 7).

The bimetal has an isotropic behavior, exhibiting properties with the same values when measured along the axes in all directions [16]. Such a uniform behavior restricts the range of movement. However, the use of such characteristics innate to bimetals result in a series of positive implications including predictability, precision, reduction of power consumption, and low maintenance [40].

The responsive potential of bimetals has been gaining relevance in the last twenty years for developments in architecture. Sung’s work is probably the most developed example of architectural skins [8]. One example is the InVert Auto-Shading Windows, which is an organic shutter system that reduces heat gain and power consumption [10]. Sung’s project uses smart geometries [17] that rotate under the effect of solar radiation, without using an electromechanical system. Other researchers associate the bimetal with non-responsive materials in their facade proposals [16]. Thus, it is possible to broaden the usage of bimetals by optimizing their performance, developing smart forms, and combining solutions with other types of materials.

## 3. Results

### 3.1. Observing Nature Study I

**Figure 8:**
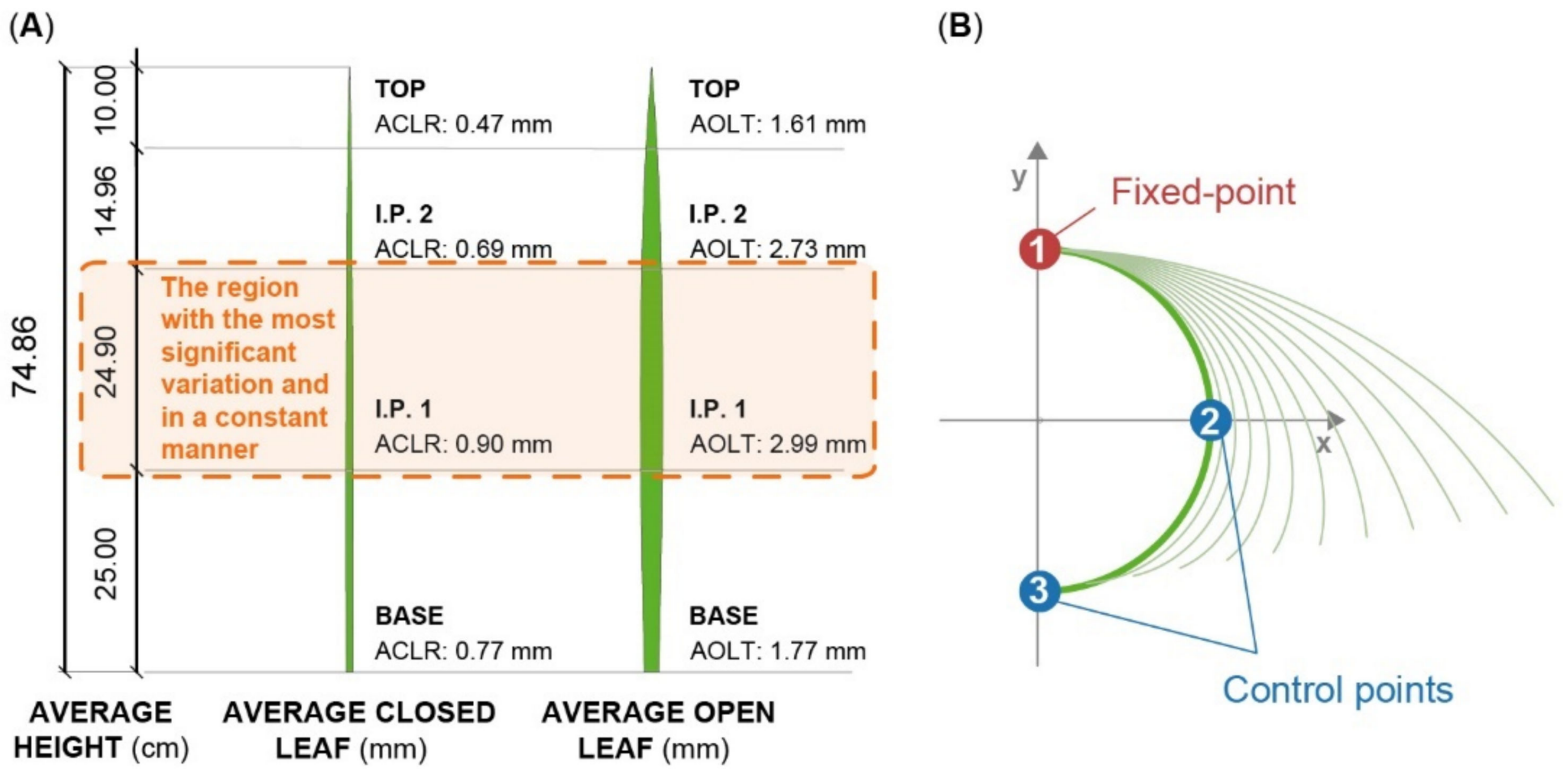
(**A**) An average leaf: height, closed and open leaf. For each of the four pre-determined points, we identified: ACLR and AOLW; (**B**) Conceptual process for defining the algorithm from (1) fixed point and (2–3) control points, which allow the leaf opening and closing. Source: Authors.

We calculated the average leaf from the collected data, referring to each of the four pre-determined points. Figure 8A shows the average height, the average closed-leaf radius (ACLR), and average open-leaf width (AOLW) for each of the four pre-determined points.

**Scheme 1:**
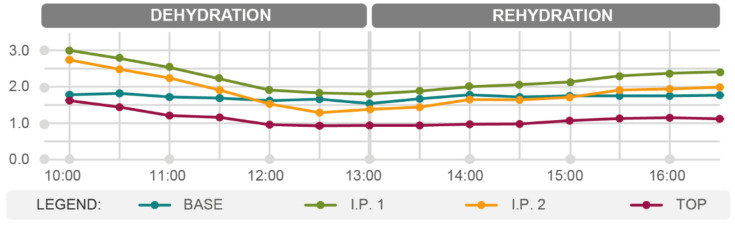
Average variation of leaf width in mm/time (min). Dehydration cycle: 10:00–13:00. Rehydration Cycle: 13:01–16:30. Source: Authors.

Scheme 1 shows the average variation of leaf width in mm/time.

From the data analysis, we could observe that the intermediate region of the leaf presents more significant variability than the extremities (Figure 8A). The dimensions of the base and top have reduced opening variations. I.P. 1 and 2 have similar leaf opening and closing behavior (Scheme 1). The dehydration process takes around three hours. Rehydration showed a longer response time than dehydration. However, the samples under analysis were not in their natural habitat.

When we compared the openings of the leaf samples with the initial measurements and with those made twenty-four hours later, we verified that all the leaves reopened. The base points showed an average increase of 0.05 mm in the initial widths of the samples. On the other hand, I.P.1, I.P.2, and Top points showed width reduction by 0.26, 0.41, and 0.16 mm, respectively. Thus, this observation study analyzed the reversible leaf movement of the *Ammophila arenaria*.

### 3.2. Analogy and Beginning Conceptualization Phase

An analogy is a cognitive process of establishing relations between two things by some kind of perceptual similarity. Hence, a biomimetic analogy is an action of interpreting nature’s references by transferring information from them to the artificial world to create artifacts with formal, functional, material, and evolutionary characteristics [1]. Therefore, it is essential to understand and capture the principle of how something happens in nature and then apply it to new knowledge [1].

The observation studies of the *Ammophila arenaria* demonstrated the organism uses different strategies that can serve as inspiration, such as:

- Leaf-rolling mechanism.
- The location of the bulliform cells in the epidermis determines the leaf closing pattern.
- Lengthwise cone-leaf closure shape, in which the ends of the base and top arch tend to meet and form a circle when closed.
- Cross-section morphology, where the adaxial side is ribbed and densely covered by hairs.

Based on these results, we used the reversible movement of the leaf-rolling mechanism, lengthwise cone-leaf closure shape, and the position of bulliform cells in analogy to *A. arenaria.* Subsequently, we will apply the knowledge acquired about *Ammophila arenaria* in the parametric leaf-blade system and experiments with the bimetal.

#### 3.2.1. Parametric Leaf-Blade System

We sought an analogy between the morphological characteristics and the behavior of the *Ammophila arenaria* to develop a parametric algorithm of the leaf movement with lengthwise cone-leaf closure shape. We used Rhinoceros 6.0 software and the Grasshopper plug-in to analyze the data collection of our five samples, based on Figure 8A.

For the conceptual process of the parametric model in Grasshopper, we first defined the circle’s central point (Figure 8B). Henceforth, the algorithm will present:

- a fixed point or starting point (located at the angle of the circle π rad).
- a control point or terminal point, located at angle 3*π/2.
- a control point at angle π/2 (which identifies the tangent vector).

Subsequently, we implemented Arc SEC to design parametric arcs as a function of the radius. We applied this logic to define the radius of I.P.1 (parameter 1: Radius A) and of I.P.2 (parameter 2: Radius B). These arcs allow us to control different degrees of leaf opening and closing (parameter 3) depending on the movement of the control points in the cartesian plane (x-y axes) (Figure 8B).

**Figure 9:**
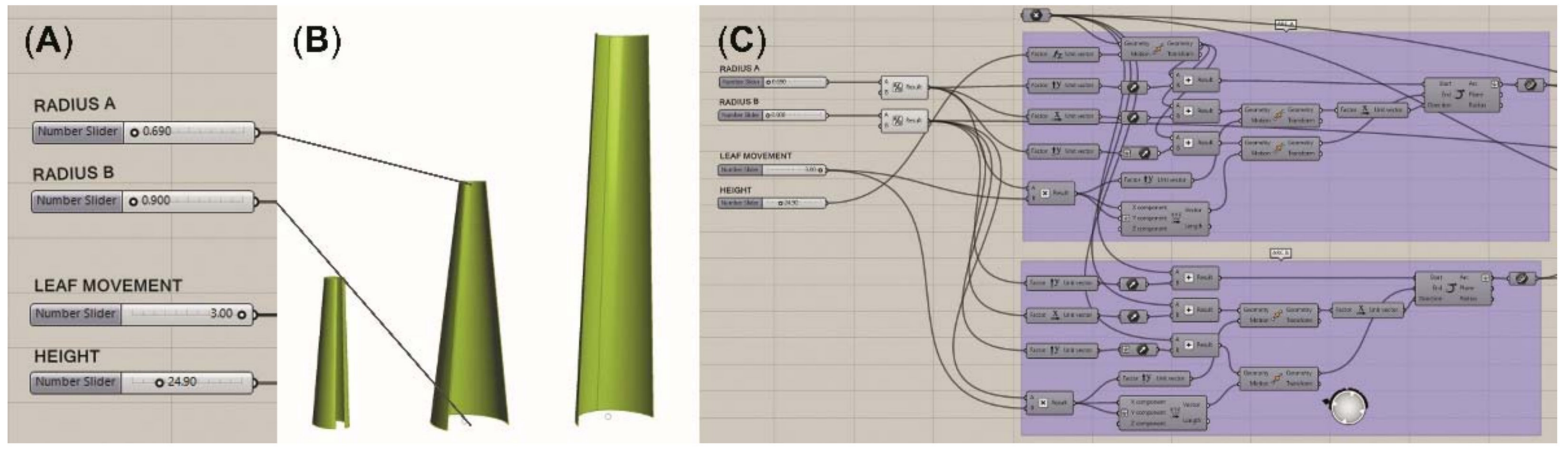
Parametric leaf-blade: (**A**) Parameters; (**B**) Different degrees of leaf blade radius, height, and opening; (**C**) algorithm. Source: Authors.

We separated the arcs at a distance on the *z*-axis (parameter 4: leaf blade height). We generated a surface between them, and therefore, created the mirroring of the surface in order to form the leaf module algorithm from the union of both surfaces. The algorithm allows the parametric modification of the leaf-blade following radius A, radius B, leaf movements, and height (Figure 9).

**Figure 10:**
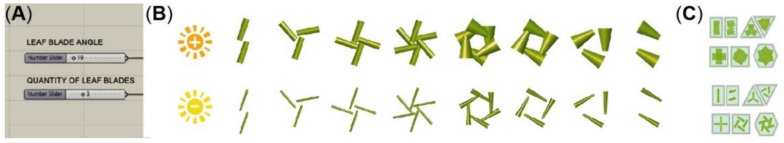
Parametric leaf-blade system: (**A**) parameters for replicating the modules of leaf-blade; (**B**,**C**) different positioning patterns, high (+) and low (−) radiation intensity. Source: Authors.

Next, we developed a code to allow for control of the position of the leaf blades equidistant to a center of a circle. We added more parameters to the parametric leaf blades: angle (parameter 5) and quantity (parameter 6). This mechanism aims to protect windows from excess solar radiation on hot days, and simultaneously, maximize solar radiation on days with mild temperatures, (Figure 10). We positioned the leaf blades between two parallel and distinct planes to avoid contact between them and allow movement.

We developed the algorithm parametric leaf-blade system in analogy to *A. arenaria*’s leaf movement with the intent of designing self-shading modules for adaptive and responsive facades.

The objective of this research step is twofold: (1) to develop a computational geometrical model capable of reproducing the behavior of the leaf movement; and (2) to be able to reuse such a model later on to simulate the behavior of facade solutions resorting to modular elements conceptually based on this behavior. The simulation step is referred to in the research plan as future work.

#### 3.2.2. Observation Study II

**Figure 11:**
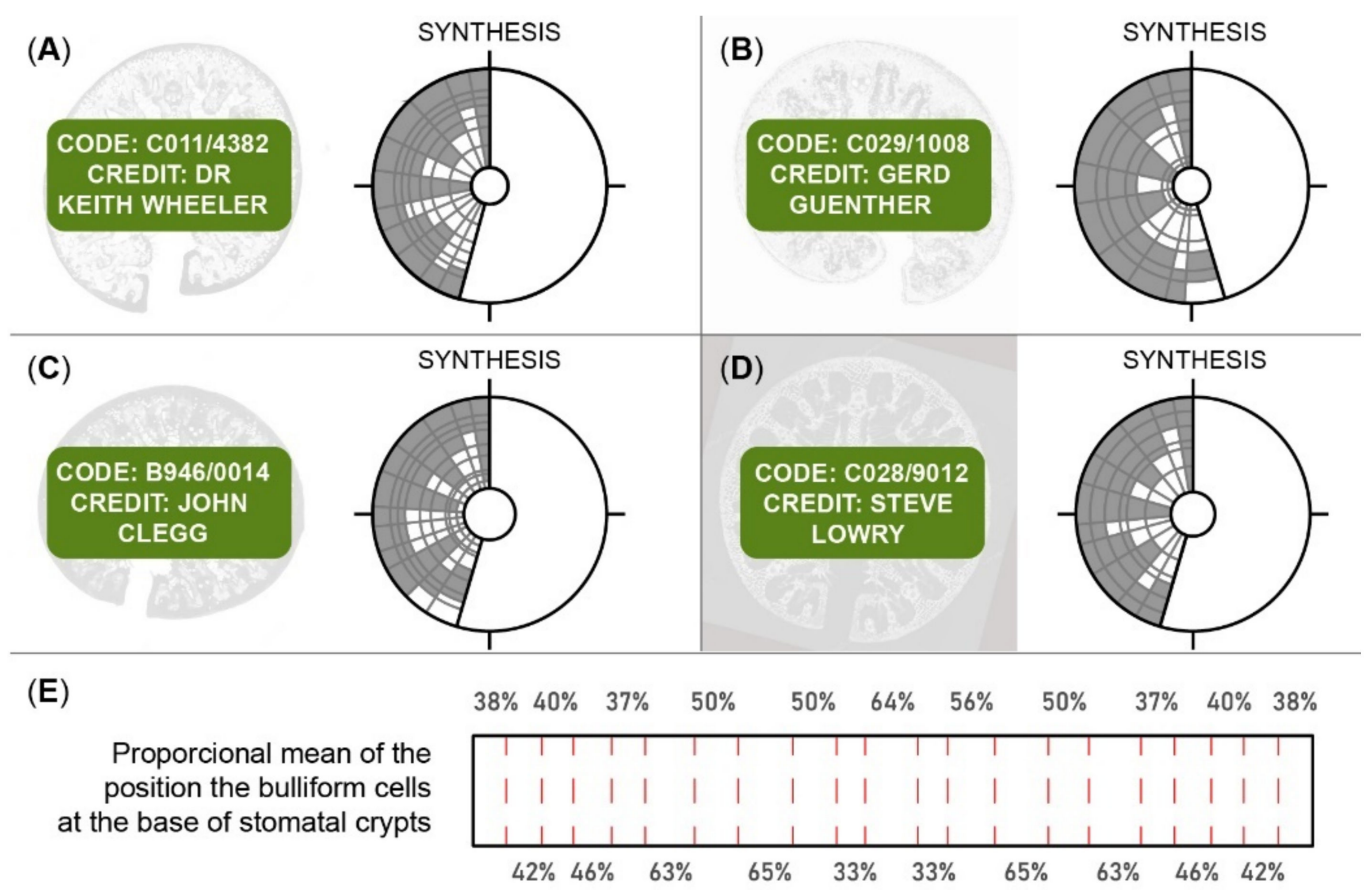
Schematic diagram of the cross-section analysis (**A**–**E**). Source: images cross-section [35]; Schematic diagram. Source: Authors.

The cross-section analysis of the *Ammophila arenaria* corresponds to the morphological analogy of the position of the bulliform cells in the base of stomatal crypts. To do this analysis, we drew a circle with a radius of 10 cm over the image of each cross-section using the Adobe Illustrator software. As we rotated the circle and radius, we marked the deepest points of the base stomatal crypts, in two quadrants of the cross-section: quadrant 1: π/2 to π rad; quadrant 2: π to 3 π/2 rad. Subsequently, we filled in the gaps according to the cross-section’s morphology. The schematic diagram (Figure 11) presents a qualitative synthesis of the *A. arenaria* cross-sections analysis.

To create the crease patterns synthesis on a 5 cm line, we traced the points of the stomatal crypts by using Rhinoceros 6.0 Software. Following this, we calculated the mean between these points for the four sections, we then duplicated them ending up with a 10 cm strip and established two pattern types:

- Standard CS20: obeys the same proportions identified in the *A. arenaria* cross-sections analysis and contains twenty markings.
- Standard CS14: an extended version of the CS20 pattern, which contains fourteen markings.

We believed the creases applied on the surface could influence the movement pattern of the shape-changing smart materials, such as the bimetal. This will be analyzed and demonstrated experimentally in the next section.

Another point we identified from the analysis was that to close the leaf, the ends of the *Ammophila arenaria*’s cross-section coincide with the leaf ends. They are parallel.

#### 3.2.3. Experiments with Bimetal

We carried out a series of experiments to assess the possibility of adapting the kinematic behavior of the bimetal to close when heating and to open when cooling, inspired by the *Ammophila arenaria*. This may be useful to improve building sustainability as shading devices for buildings are crucial to maximizing solar radiation in winter (low temperatures) and to providing protection from solar radiation during summer (high temperatures) [11,36].

The material’s behavior can be optimized, enhanced, or canceled depending on its shape [17]. The experiments aimed to comprehend, adapt, and control the material’s kinetic capacity in different temperatures, varying its shape and creasing patterns on its surface. We use the bimetal range with the PR675r (0.0025″ thick and 100 mm) technical specification. The high expansion layer is composed of Mn75Ni15Cu16 and the low expansion layer of Ni36.

**Figure 12:**
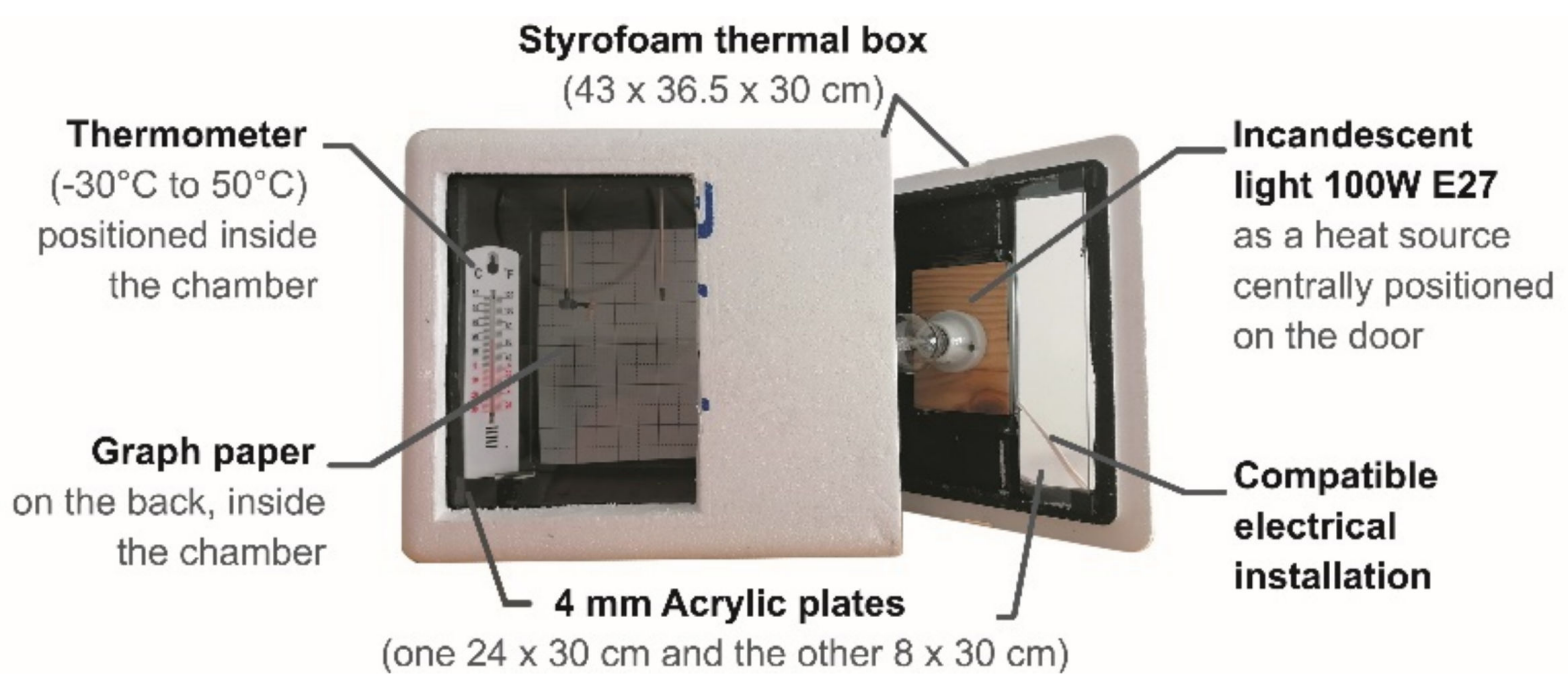
Hot chamber. Source: Authors.

To evoke the bimetal thermo-responsive performance, we constructed a hot chamber that simulates climatic thermal variation (room temperature to 50 °C) (Figure 12). The chamber allowed a heat supply without airflow, which enabled us to easily fixate and test different samples, and switch it on and off.

To initiate the experiment in the hot chamber, we placed the samples inside and fixed the mobile phone to film using a stand, facing the chamber’s front window. Once we started recording, we collected the local climatic data at the starting time of the experiment: room temperature, relative humidity, atmospheric pressure, and wind speed.

We registered the temperature as it changed in the chamber, the time of the video, and notes on the deformation of the material. We later analyzed the footage corresponding to each registered temperature and time. Finally, we treated and synthesized the material’s behavior at different temperatures.

The initial exploratory tests made it evident that the optimization of bimetal deflection depends on how the material is cut concerning its width [38]. That is, sample A (100 × 20 mm) will present curvature to the passive side of the layer at 30 °C. This deflection will be greater than sample B (20 × 100 mm). For this reason, we chose to explore the development of the samples towards the width of the material.

At first, in an exploratory mode, we manually created creasing patterns on the passive and active sides. If the creases are generated with a subtractive material process, the depth of the creases can weaken the material and compromise its durability due to repetitive movements in response to environmental conditions. The ideal solution is to create creases as an effect from folding the material, with an angle smaller than 90°.

We noticed the potential to explore the crease on the active layer once the initial evidence demonstrated an opportunity to manipulate the kinematic behavior of the bimetal. In other words, the crease patterns can lead to an open form when heating and closed when cooling.

In the following section, we will describe the three initial experiments that demonstrated this adaptation and aim to expand the kinematic limits of bimetal with regards to our base experiment, experiment variants, and experiment with lengthwise versions. After preparing the samples, we observed their behavior in the hot chamber at different temperatures.

**Figure 13:**
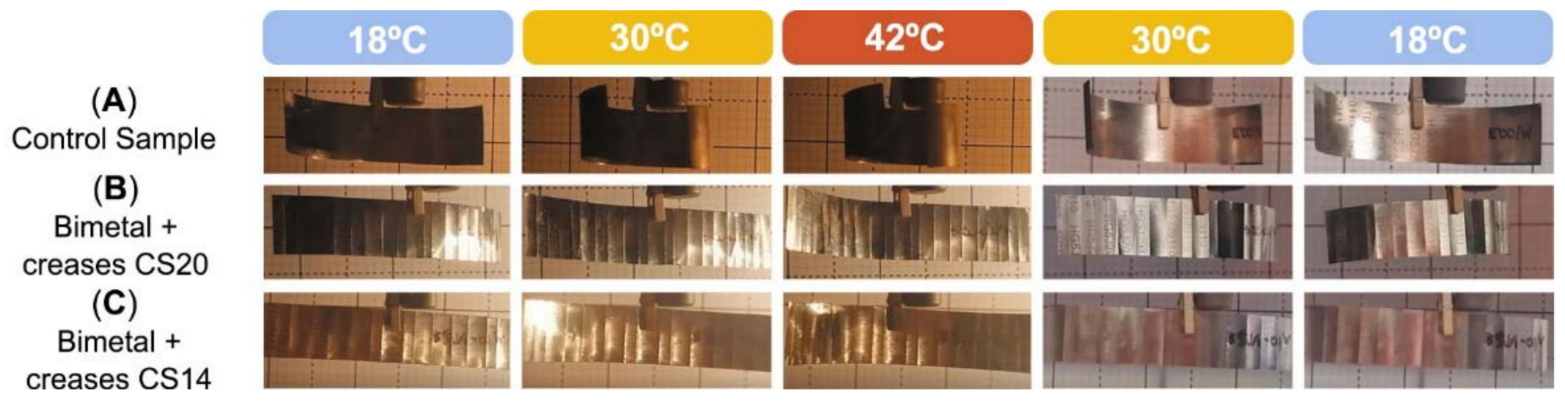
Base experiment: samples submitted to different temperatures (**A**–**C**). Source: Authors.

For the ‘base experiment’, we initially made samples in the same proportion (100 × 20 mm) using different patterns: (a) control sample with no intervention (under heat effect this sample presents deflection towards the passive layer); and bimetal samples with crease patterns (b) CS20, and (c) CS14 (observation study III). Samples CS20 and CS14 had creases perpendicular to the width under the active layer. Figure 13 compares the behavior of the samples in different temperatures in the hot chamber.

As we fixed the crease patterns, the samples tended to become more rectilinear or even reverse to the initial angle towards the negative quadrants (i.e., towards the active side of the bimetal). Consequently, the effect of the creases changes the initial angles of the material, with the deformation facing the active layer, instead of the passive one.

The closing and opening process reversibly occurs as an immediate response to the thermal impact on the bimetal’s surface. That is, if we apply a constant temperature variation, the deflection will be gradual and tends to be constant. Whereas, if the temperature change is abrupt, the deflection response will be faster.

**Figure 14:**
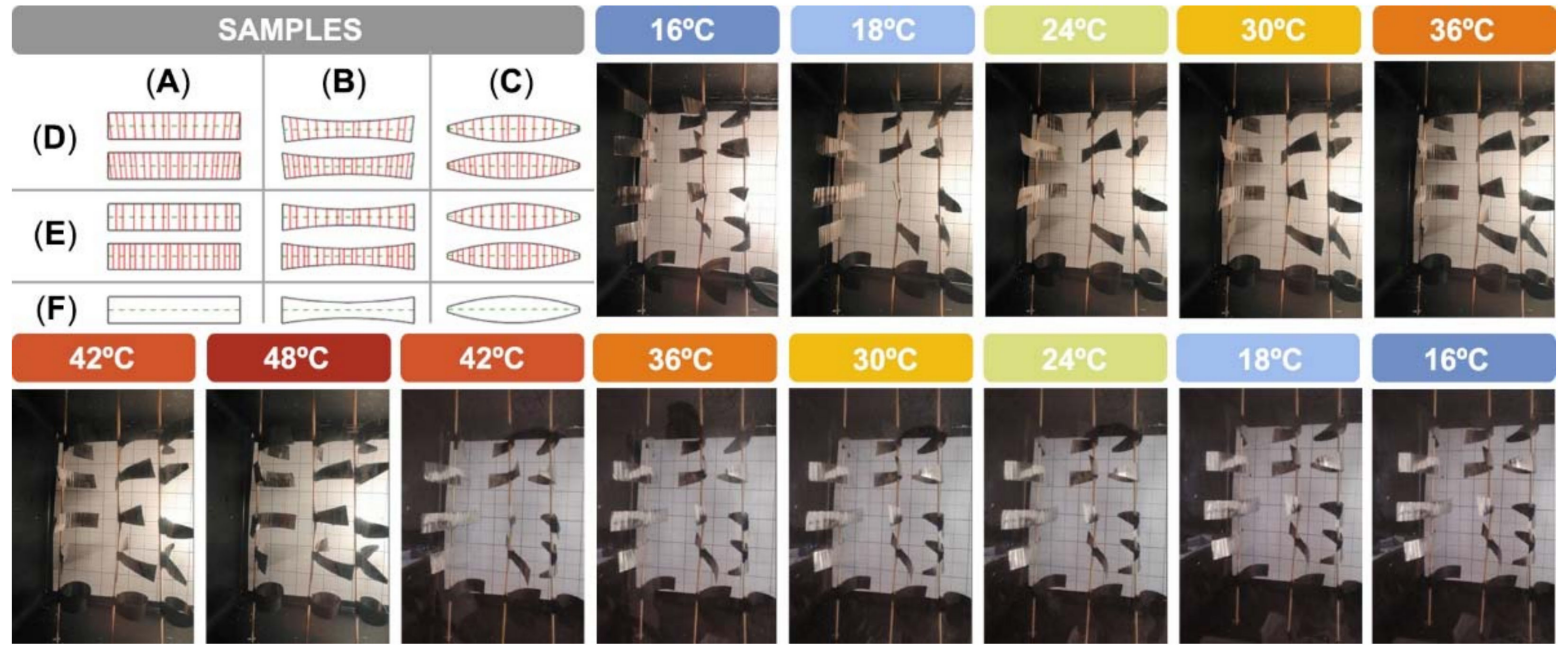
Experiment variants subjected to different temperatures. The bimetal samples were on a wooden support, as in (**A**–**C**). Sample table lines: different sample types: (**D**) oblique crease; (**E**) perpendicular crease; (**F**) control sample. Source: Authors.

In the second experiment, the ‘variants experiment,’ we observed the simultaneous reaction of different sample versions (Figure 14) in the hot chamber. The samples had the reference dimension (100 × 20 mm); different shapes, and, when applicable, the use of two types of crease inclination: different degrees of angulation (Figure 14D) and perpendicular to the width (Figure 14E).

To make the oblique creases, using Rhinoceros, we positioned the four pre-determined points to the crease patterns inspired by the *Ammophila arenaria.* We defined the creases’ angles by adopting a method that consists of establishing points and lines as references on the samples and changing the unidirectional scale factor. We used two inclination patterns: one for the CS20 and the other for the CS14. Subsequently, we applied these patterns to different 100 × 20 mm rectangle shapes.

We fixed the samples on wooden supports and placed them in the hot chamber. We performed two different sessions, rotating the fixation axes at 180°. We observed that the samples located in the upper part showed a greater variation in behavior than those located in the lower part. This occurred due to differences in air density inside the chamber. Figure 14 shows the session in which the control samples were at the bottom of the chamber. The control samples (Figure 14F) are naturally curved, the heat accentuated the movement towards the passive layer—already inclined to curving and towards the positive quadrants of the movement.

When the samples with creases were subjected to higher temperatures, their dynamic behavior transitioned from the negative (towards the active layer) to positive quadrants (towards the passive layer as it cools down). We noticed that the samples with a 20 mm height tended to have their resistance exceeded from 24 °C onwards (Figure 14). That is, the samples returned to the original direction of the bimetal deformation, towards the passive layer.

**Figure 15:**
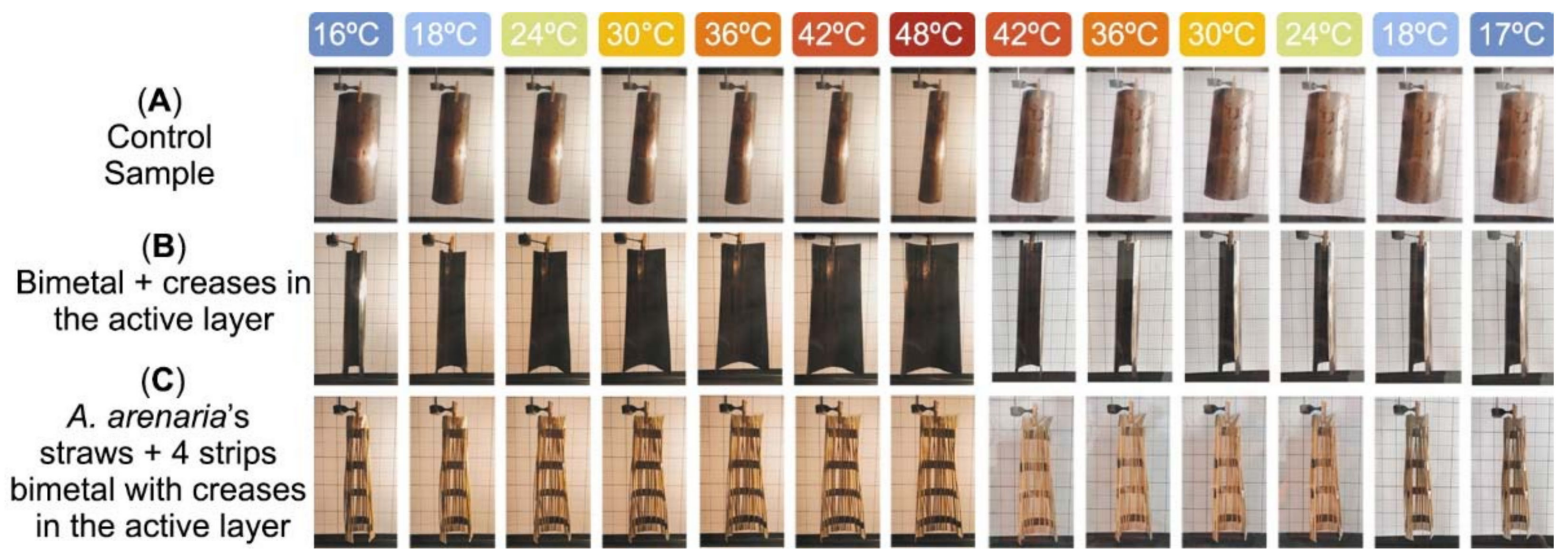
Experiment with 100 × 150 mm samples (**A**–**C**). Source: Authors.

The ‘experiment with lengthwise versions’ (Figure 15) did not show the resistance to be exceeded, and demonstrated that the height of the metal pieces directly influenced the movement performance. If the sample heights increase, it is possible to restrict the amplitude of the movement only to the side in favor of the active layer. In other words, the samples open when heated and close when cooling. In this way, it is possible to develop forms that open with heat instead of closing. Figure 15 displays an experiment with three different types of samples with 100 × 150 mm: (a) control sample; (b) bimetal with creases in the active layer (type CS20); and (c) straw structure of *A. arenaria* intertwined with four bimetal strips (10 × 100 mm with creases in the active layer, type CS20).

We found that the kinetic response to the thermal impact on the bimetal surface is immediate in the three systematized experiments. In Figure 15B, we could identify the moment the hot chamber is opened, because the sample showed a fast closing movement when cooling from 48 °C to 42 °C, demonstrating the material’s sensitivity to variations in temperature.

We do not recommend using small heights for the samples, as the repetitive movement effect in both directions—from the active to the passive side of the bimetal, and passive to active—may cause damage. Ideally, we should use creases to restrict the range of motion only depending on the active layer. We did not identify any damage to the samples with greater heights (Figure 15).

Although the adaptive response of the bimetal sample with creases in the active layer (Figure 15B) presents greater sensitivity to the temperature for opening, the straw sample from *A. arenaria* attached to four thermobimetal strips provides a similar effect (Figure 15C). One promising alternative is combining the bimetal with static and biodegradable materials. Solutions like these can enhance sustainability, not only regarding thermal responsiveness as it minimizes power consumption in built environments, but also by reducing the amount of bimetal necessary for their construction.

## 4. Discussion

We used the reversible movement of the leaf-rolling mechanism, lengthwise cone-leaf closure shape, and the position of bulliform cells in analogy to *Ammophila arenaria.* Concerning the first two, we applied the parametric algorithm, which allowed us to obtain a cone with a perfect closing system, in line with the parametric height variation and the radii. Then, we implemented lengthwise versions of the responsive modules. The parametric leaf-blade system, in turn, demonstrated its conceptual potential to be applied on facades enabling a broad aesthetic variation and different performances concerning protection from solar radiation.

Regarding the bulliform cells, we analyzed and abstracted their location. In this sense, the series of systematized experiments comprised the research on control and manipulation of the thermobimetal kinetic behavior. The experiments Base, variants, and lengthwise versions demonstrated that it is possible to create resistance to the material’s natural movement, thus allowing an opening behavior when heated and closing as it cools. In other words, it is possible to reverse the direction of the bimetal movement. To this end, the implementation of crease patterns on the active layer was crucial to obtain these results. They create resistance and adapt the materials’ thermosensitive behavior autonomously and without any electromechanical control. Furthermore, the creases on the bimetal provide us with a behavior inspired by the *A. arenaria*, under the approach of solar radiation control and thermal variation.

We consider these experiments as the starting point of the investigation, which aims to identify parameters to control the kinetic behavior of bimetal. For such, we emphasize the need to deepen the knowledge on the material through experimental and exploratory research, on:

- Crease patterns: with experiments that take into account different amounts of creases and at different angles.
- Performance of creases: testing the material’s limitations, how far we can expand its thermo-responsive performance and minimize its fragility. Consequently, we must analyze the bending process to form the crease; the productive process—manual and digital, and fatigue resistance test —to prevent unexpected behavior.
- Performance of the height of the modules: to restrict the movement towards the active side of the layer, and so providing control of the material’s behavior for opening when heated and closing when cooling.
- Combination of the bimetal with static and responsive materials: to find solutions that reduce both the use of non-biodegradable materials and power consumption in built environments.
- Smart geometries: In line with the creases in the active layer, explore complex shapes to optimize the bimetal deflection and develop a formal solution based on performance-based design. Finally, future works will propose a shading system for the facade that contributes to optimizing thermal comfort and natural lighting.

So far, the experimental evidence allows us to conclude that the crease pattern inspired by the *Ammophila arenaria*, applied on the bimetals active surface, directly influences the materials behavior. Another conclusion refers to the sample’s height as crucial to avoid possible damages to its structure because of repetitive bidirectional movements and the thermoresponsive movement’s performance. Therefore, restricting the material’s movement only towards its active layer is essential to promote lighting control and protection from the excess of sun radiation.

Further research and experimentation will be required to fully understand and have control over the material’s behavior. We intend to identify the ideal relationship between creases on the active side of the bimetal layer, height, and geometry to increase the performance of the facade responsive modules. We hope to use these parameters to adjust and customize the material’s thermoresponsive behavior to promote protection from sunlight, natural lighting, and provide privacy to built environments.

## 5. Conclusions

This study evidenced the sophisticated adaptive capacity of *Ammophila arenaria* to climate conditions such as humidity. Additionally, the bio-inspiration by this organism can transcend approaches that are aimed solely at the hydromorphic property. We demonstrated that it is possible to apply the strategies of *A. arenaria* to shape-changing smart materials and reverse their behavior as we did with the bimetal. The creases in the active layer disclosed a high design potential for developing shading facades in response to thermal variation and solar radiation. Once having a rigorous understanding and control over the material behavior, we plan to continue this research to develop an adaptable and responsive cobogó, inspired by *Ammophila arenaria.* In the future, we shall integrate the modules’ responsive behavior, developing the parametric algorithm and climatic simulations to optimize the solution. Furthermore, such facade concepts may contribute to the control of heat gain in the built environment without requiring any electromechanical devices.

## Data Availability

The data presented in this study are available on request from the corresponding author.
