# Correction: Andrade et al. The Adaptive Power of *Ammophila arenaria*: Biomimetic Study, Systematic Observation, Parametric Design and Experimental Tests with Bimetal. *Polymers* 2021, *13*, 2554

**DOI:** 10.3390/polym15020366

**Published:** 2023-01-10

**Authors:** Tarciana Araújo Brito de Andrade, José Nuno Dinis Cabral Beirão, Amilton José Vieira de Arruda, Cristina Cruz

**Affiliations:** 1 Centro de Investigação em Arquitetura, Urbanismo e Design (CIAUD), Faculdade de Arquitetura, Universidade de Lisboa, 1349-063 Lisboa, Portugal; 2 Programa de Pós-Graduação em Design (PPGDesign), Universidade Federal de Pernambuco, Recife 290, Brazil; 3 Department of Plant Biology, Faculdade de Ciências da Universidade de Lisboa, 1749-016 Lisboa, Portugal

## 1. Update on the Acknowledgments

In the previously published paper [1], there was no information about FCT funding through the Strategic Project referenced below. We updated the information to:

**Acknowledgments**: This work is financed by national funds through FCT—Fundação para a Ciência e a Tecnologia, I.P., under the Strategic Project with the references UIDB/04008/2020 and UIDP/04008/2020, and through the scholarship SFRH/BD/144910/2019.

## 2. Text Correction

There was an error in the original publication. For the 10 cm-wide piece of bimetal under analysis, the natural behavior is to open when cooling and close when heated. When we insert the creases on the active layer, the behavior is the opposite— it closes when cooling and opens when heated.

A correction has been made to **3.2.3. Experiments with Bimetal**: “We noticed the potential to explore the crease on the active layer once the initial evidence demonstrated an opportunity to manipulate the kinematic behavior of the bimetal. In other words, the crease patterns can lead to an **open** form when heating and **closed** when cooling.”

Regarding the scientific interpretation, there is no change. This replacement is to be more explicit about the results. The authors apologize for any inconvenience caused and state that the scientific conclusions are unaffected. This correction was approved by the Academic Editor. The original publication has also been updated.
